# Has C-Peptide Come of Age?

**DOI:** 10.1155/EDR.2001.81

**Published:** 2001

**Authors:** Anders A. F. Sima


### Editorial: Has C-Peptide Come of Age?

In this issue, a Consensus Statement on proinsulin C-peptide is published summarizing the deliberations of an international group of investigators, who met in Detroit in October 2000. This was preceded by the 3rd International Motor City Symposium, the theme of which was concerned with the clinical and biological effects of C-peptide. The abstracts of the presentations at the Symposium are also published in this issue of the International Journal of Experimental Diabetes Research.

There is no doubt that the understanding of the actions of C-peptide has come a long way since its original discovery in the late 1960s.[1] A biological effect of C-peptide was suspected at the time. It was hypothesized that C-peptide would have an insulin-like glucose lowering effect. However, several studies failed to demonstrate such an effect and it was concluded that the only role played by C-peptide was in the stereometric formation of insulin and that it lacked further biological activities.

In the early 1990s, the interest in C-peptide was revitalized by Dr. John Wahren and his group at the Karolinska Institute in Stockholm.[2] In short time C-peptide replacement studies in type I diabetic patients demonstrated beneficial effects on incipient nephropathy with partial reversal of glomerular filtration rate and albumin secretion. Beneficial effects were also demonstrated in type I patients with autonomic and sensory neuropathies. These findings were paralleled by effects on skin and muscle blood flow. These encouraging data energized the search for potential mechanisms, which followed with the demonstration of corrective effects on neural and renal tubular $Na^+/K^+$-ATPase and microvascular nitric oxide. These findings appeared, at least in part, to explain the clinical effects. A further milestone in the C-peptide saga was the demonstration of its specific binding to cell membranes[3] by Riegler and collaborators at the Karolinska Institute. These findings strongly suggest a receptor-mediated mechanism for C-peptide's action. Studies addressing the characterization of a specific C-peptide receptor are ongoing at the Karolinska Institute. Simultaneous studies at the Wayne State University/Morris Hood Diabetes Center in Detroit suggested an alternative and/or adjunct mechanism for C-peptide. Grunberger and collaborators[4] demonstrated activation of the insulin signaling pathway by C-peptide alone and with additive effects when incubated together with insulin. These results were supported by the finding that C-peptide autophosphorylates the insulin receptor. Since C-peptide does not compete with insulin at the receptor level, these findings may suggest a different ligand site for C-peptide. The receptor issue, particularly that of a specific C-peptide receptor, is not settled and remains one of the key issues to be firmly established, as stated in the recommendations by the Consensus Statement.

The enormous potential benefit of C-peptide in clinical medicine undoubtedly lies in its probable effects in preventing and ameliorating the chronic complications in type I diabetes, which account for the high morbidity and mortality in this patient group. Long-term experimental studies have demonstrated significant preventive and interventional effects on diabetic neuropathy[5], which is more severely expressed in type I as compared to type II diabetes. Another complication that is being increasingly recognized clinically is a duration-dependent cognitive deficit in type I diabetic patients which is unrelated to hypoglycemic episodes. There are experimental data from the Detroit Center demonstrating a duration-dependent programmed neuronal cell death in brain areas associated with cognitive function and encouraging results showing a significant protective effect following C-peptide replacement on type I diabetic rats.

In summary, both clinical and experimental data to date are promising and further explorations of the biological effects of this small peptide should be encouraged as outlined by the Consensus Group. In lieu of any effective treatment for most of the devastating chronic complications in type I diabetes, C-peptide holds great promise as an effective, inexpensive and simple therapy and, unlike some of the evolving alternatives, is not likely to cause adverse clinical effects.

### References

1. Steiner, D., Cunningham, D., Spigelman, L., and Alen, B. (1967): Insulin biosynthesis: evidence for a precursor. Science, 157, 697-700.
2. Johansson, B-L., Linde, B., and Wahren, J. (1992): Effects of C-peptide on blood flow, capillary diffusion capacity and glucose utilization in the exercising forearm of type I (insulin-dependent) diabetic patients. Diabetologia, 35, 1151-1158.
3. Rigler, R., Pramanik, A., Jonasson, P., et al (1999). Specific binding of proinsulin C-peptide to human membranes. Proc. Natl. Sci. USA, 96, 1318-1323.
4. Grunberger, G., Qiang, X., Li, Z.-G., et al (2001): Molecular basis for the insulinomimetric effects of C-peptide. Diabetologia, (in press).
5. Sima, A.A.F., Zhang, W., Sugimoto, K., et al (2001): C-peptide prevents and improves chronic type I diabetic neuropathy in the BB/Wor-rat. Diabetologia (in press).

*Anders A.F. Sima*

